# What Drives Employees' Innovative Behaviors in Emerging-Market Multinationals? An Integrated Approach

**DOI:** 10.3389/fpsyg.2021.803681

**Published:** 2022-01-20

**Authors:** Shanyue Jin, Yannan Li, Shufeng Xiao

**Affiliations:** ^1^College of Business, Gachon University, Seongnam, South Korea; ^2^Graduate School of Technology Management, Kyung Hee University, Yongin, South Korea; ^3^Division of Business Administration, Sookmyung Women's University, Seoul, South Korea

**Keywords:** Chinese MNCs, innovative behavior, organizational climate, psychological stability, servant leadership, work-life balance

## Abstract

The coronavirus disease 2019 (COVID-19) pandemic has severely damaged the global industrial supply chain and accelerated the digital transformation of the global economy. In such rapidly changing environments, multinational corporations (MNCs) should encourage employees to be more innovative in various fields than ever before. With the onset of the COVID-19 pandemic, employees have become psychologically anxious, their working conditions have deteriorated, and they are in danger of losing their jobs. In this study, we aim to address the question of whether servant leadership facilitates the innovative behavior of employees working in emerging-market MNCs when servant leadership is adopted within the firms. In addition, we explore the mediating roles of work–life balance and psychological stability perceived by employees, and the moderating role of organizational climate in the relationship between servant leadership and MNC employees' innovative behavior. In doing so, we collected data from a sample of 307 Chinese employees who are employed by five different Chinese MNCs from the Internet, information technology, electronics, and e-commerce industries. Based on a sample of survey data collected from employees of Chinese MNCs, we empirically test these ideas by specifically examining how servant leadership may shape the innovation behavior of employees in these MNCs. The results suggest that servant leadership positively influences employees' innovative behavior, and that the contribution of servant leadership to employees' innovative behavior is mediated by work–life balance and psychological stability as well as moderated by the degree of organizational climate. Moreover, the different organizational climates of these MNC employees are also expected to significantly shape the relationship between servant leadership and employees' innovative behavior. This study enriches our understanding of the importance of servant leadership in driving the innovative behaviors of employees in emerging-market MNCs and provides new insights into the mechanisms through which emerging-market MNCs can motivate their employees to be more innovative in their jobs. Thus, this study contributes to the research on human resource management by offering important implications vis-à-vis how MNCs manage their employees more effectively in addressing and responding to the dramatically changing global landscape in the post COVID-19 era.

## Introduction

The effects of the coronavirus disease 2019 (COVID-19) pandemic on humans cannot be overemphasized. Major economic, business, and industry impacts have been felt globally, and it has also accelerated enterprises' quick understanding of the value of digital transformation (Olokundun et al., 2021). The ever-changing business environment forces firms to view innovation as a source of productivity, efficiency, and sustainability (Hermundsdottir and Aspelund, 2021). Given the increased pressure to compete with both emerging- and developed-market multinational corporations (MNCs) in the international market, MNCs must improve their innovation in both developed and emerging markets (Su et al., 2021). However, in emerging markets, which usually imitate advanced business models compared to those in developed countries, the MNCs' innovation incentive system in these economies is not perfect (Duan et al., 2021). Therefore, MNCs should encourage employees to enhance their innovativeness in various fields. Employees' innovative behavior in an organization results from their intention to generate new ideas, processes, and procedures (Karatepe et al., 2020). Leaders are the core of an enterprise, and they play a vital role in its development. Therefore, scholars have conducted considerable research on how to improve the effectiveness of leadership and stimulate employees' innovative behaviors (Gil et al., 2018). The effective leadership style for today's dynamic environment is servant leadership (Eva et al., 2019). Servant leadership enhances employee creativity and innovative behavior (Dierendonck and Nuijten, 2011).

Servant leadership is defined as leadership that regards service to employees as a source of influence (Greenleaf, 1977). It helps employees to achieve their objectives, who, in turn, assist the firm to realize its goals. Studies proving the positive role of servant leadership in enhancing employees' innovation behaviors are increasing (Faraz et al., 2019; Hale et al., 2020; Karatepe et al., 2020). However, the underlying cognitive processes through which servant leadership triggers employees' innovative behaviors are underexplored (Eva et al., 2019). Certain studies have investigated the influence of servant leadership on employee creativity and innovative behaviors by creating a service culture (Liden et al., 2014), psychological empowerment (Faraz et al., 2019), job autonomy, and meaningful work (Cai et al., 2018). In compliance with the social distancing regulations imposed by national governments to inhibit the spread of the COVID-19-virus, many employees have continued their regular work activities while working remotely using information and communication technologies (Coun et al., 2021). The new work model and environment will also address employees' work–life balance (WLB) and psychological safety issues. A servant leader satisfies the members' basic needs, particularly psychological needs, as well as those needs that determine employees' creativity (Van Dierendonck, 2011; Sidani and Rowe, 2018). In this study, we expect that psychological safety and WLB play an important role in the relationship between servant leadership and employees' innovative behaviors. Psychological safety refers to a shared belief among work unit members that it is safe for them to engage in interpersonal risk-taking (Edmondson, 1999). Employees' psychological safety is met when they feel that taking risks and offering novel ideas are safe. The psychological safety of the members mediates the impact of the leader's servant leadership on employees (Iqbal et al., 2020).

Many studies have documented how a lack of WLB can result in deleterious effects on psychological and physical well-being as well as increased family and marital tensions (Frone et al., 1994; Lewis and Cooper, 1999). Human resources are a major source of innovation in organizations. In addition, WLB is an effective means of human resource management (Alegre and Pasamar, 2018). A high level of WLB in an organization enables workers to control their performance, such as allocating their working time efficiently. They also feel motivated, thus promoting their learning and innovation skills (Ko et al., 2020). Servant leadership helps to maintain a good WLB for employees, and this is crucial to their health and organizational success (Hale et al., 2020).

Individual creativity and leadership can be increased in a risk-taking-supported organizational climate (Wang and Rode, 2010). High congruence between a creative person and culture may increase innovative performance levels (Amabile, 2000). Organizational climate is behaviorally oriented, that is, climates for creativity, innovation, and safety represent the perceptions of organizational humanism, policies, practices, procedures, formalization, risk-taking, and subsequent patterns of interactions and behaviors. These perceptions support creativity, innovation, safety, and service in an organization (Schneider, 1990; Patterson et al., 2005; Ötkena and Cenkcib, 2015).

In this study, we aim to address the question of whether servant leadership facilitates employees' innovative behavior in emerging-market MNCs when servant leadership is adopted by firms. In addition, we explore the mediating roles of WLB and perceived psychological stability of employees and the moderating role of organizational climate in the relationship between servant leadership and MNC employees' innovative behavior. Thus, this study contributes to research on human resource management by offering important implications regarding how MNCs can manage their employees more effectively in addressing and responding to the dramatically changing global landscape in the post COVID-19 era. In particular, this study demonstrates the importance of servant leadership to ensure the psychological safety of employees at work and to improve their WLB during the epidemic. Furthermore, the climate of the organization creates robust conditions for employees to innovate. The outcome will therefore provide an academic reference value for future research on servant leadership.

## Theoretical Background and Hypothesis Development

### Servant Leadership

Since 2004, research on servant leadership has increasingly been published in high impact factor journals. However, numerous articles on servant leadership still appear in second-tier leadership journals (Eva et al., 2019). Servant leadership is a moral form of leadership that differs from other leadership styles, such as transformational, ethical, and authentic leadership (Iqbal et al., 2020). Servant leadership is more focused on the psychological needs of followers as a goal in itself, whereas transformational leadership places these needs secondary to the organization's goals (Dierendonck et al., 2014). In its leadership focus, servant leadership is followers first, organizations second, and their own interests last (Eva et al., 2019). Similarly, Greenleaf (1977) also states that “the servant leader is servant first. It begins with a natural feeling that one wants to serve, to serve first. Then conscious choice brings one to aspire to lead” (Greenleaf, 1977; Wang et al., 2019). Similar to authentic leadership, servant leadership is self-aware, empathetic, and authentic (Greenleaf, 1970; Sidani and Rowe, 2018). However, the authentic leader's creed must be “be myself.” Conversely, servant leaders are focused on listening, promoting healing and wholeness, serving others, commitment to stewardship, building community persuasively, good foresight, conceptual skills, and so on (Greenleaf, 1970). Servant leadership behaviors include the decision to serve, service tenure, concern for others' altruism, prioritizing other people, humane humility, gratitude, forgiveness, patience, compassion, justice, trust in self and others' moral honesty, integrity, fairness, seeking ethical behavior, acceptance of feedback, reflective, or philosophy greater than oneself, respect for and differences, and so on (Hale et al., 2020). It focuses on developing employees to their greatest potential in task effectiveness, community stewardship, self-motivation, and future leadership capabilities (Liden et al., 2014). Liden et al. (2014) define servant leaders' focus on providing tangible and emotional support to their followers, which helps employees to maximize their potential. Employees expect servant leaders to provide them with their needs.

Servant leadership's commitment, trust, and work–family balance provide a work climate for sharing family concerns, organizational identification, work engagement, psychological empowerment, and ensuring psychological safety (Zhang et al., 2012; Krog and Govender, 2015; Chughtai, 2016; Haar et al., 2017; Faraz et al., 2019; Utama et al., 2021). Additionally, servant leadership helps to improve individual and unit performance as well as encourage innovative work behaviors (Liden et al., 2014; Handayani et al., 2021). However, although the servant leadership construct is well-conceptualized in the literature and seems to provide favorable individual, team, and organizational results, research on the effective implementation thereof is still in progress (Coetzer et al., 2017).

### Servant Leadership and Innovative Behaviors

Leadership style positively influences the progress of innovative behaviors (Mansoor et al., 2020), such as empowering leadership, directive leadership (Coun et al., 2021), and participative leadership (Chen et al., 2020), especially servant leadership (Erkutlu and Chafra, 2015). Organizations need employees to cope with the changing and complex environment, and, thus, continuously improve their innovative behaviors. Innovative behavior refers to introducing, developing, and implementing new ideas to provide useful and novel solutions to help organizations to solve problems (Scott and Bruce, 1994). Innovative behavior includes three stages: idea generation, promotion, and realization (Scott and Bruce, 1994; Janssen, 2000).

Erkutlu and Chafra (2015) also mention that servant leadership is an element that builds organizations' innovation behavior. A good application of servant leadership positively impacts innovation implementation behavior (Putri and Utama, 2018). Servant leadership can create a climate that encourages employees to behave innovatively (Handayani et al., 2021). Jan et al. (2021) state that servant leadership positively influences employees' innovative work behavior. Therefore, we predict the following relationship:

*Hypothesis 1*: Servant leadership has a positive influence on innovative behavior.

### Servant Leadership and Work–Life Balance

Haar (2013) defines WLB as “the degree to which an individual can adequately manage multiple roles in life, including work, family, and other major responsibilities.” He believes that WLB affects work and happiness. In this context, Haar et al. (2017) defines WLB as an individual's ability to meet work and family commitments, as well as non-work-related responsibilities and activities. Notably, WLB can help both companies and employees to manage their family responsibilities, create flexible work conditions, and enable them to perform better, particularly in MNCs (Pradita and Franksiska, 2020).

Servant leadership has been proven to be an important source of work-related resources for employees who aim to improve their family lives (Zhang et al., 2012). However, studies on the relationship between servant leadership and WLB (Utama et al., 2021). Workplace factors are important to WLB, which positively impacts employees' well-being and positive energy at work (Russo et al., 2016). Effective human resource management supports employees in satisfying their WLB through servant leadership. Conversely, employee performance will improve, thereby enhancing leaders' focus on increasing their followers and employees' WLB (Setyaningrum and Pawar, 2020). Therefore, we propose the following relationship:

*Hypothesis 2*: Servant leadership has a positive influence on work–life balance.

### Servant Leadership and Psychological Safety

Psychological safety refers to one's belief about the workplace that taking interpersonal risks, sharing ideas and opinions, and acting independently on crucial decisions are safe (Edmondson, 1999; Brohi et al., 2018). Kahn (1990) studies psychological safety at the individual level and reports that it affects individual behavior and internal motivation. Psychological safety involves more than perceiving and experiencing high levels of interpersonal trust; it also describes a work climate characterized by mutual respect (Walumbwa and Schaubroeck, 2009; Hu et al., 2018). Servant leadership encourages a positive climate wherein followers feel accepted and respected. Such a constructive relationship provides a context whereby followers perceive that developing novel ideas that are against the norm is safe (Oldham and Cummings, 1996; Yoshida et al., 2013). The literature shows that various leadership styles positively impact employees' psychological safety. These leadership styles include humble (Zhang and Song, 2020), authentic (Nielsen et al., 2013), change-oriented (Detert and Burris, 2007), and inclusive (Carmeli et al., 2010) leadership. They allow followers to feel psychologically safe to take interpersonal risks and speak out to realize their potential and growth. Thus, we propose the following relationship:

*Hypothesis 3*: Servant leadership has a positive influence on psychological safety.

### Mediating Effect of Work–Life Balance

Haar (2013) finds that WLB mediates the relationship between work and family factors (conflict and enrichment) toward the achievement of work and well-being outcomes. Moreover, WLB implicates employees' attitudes, behaviors, and well-being, as well as the organization's effectiveness (Au and Ahmed, 2014). An imbalance between work and family owing to excessive workload or family problems can result in stress and negative work attitudes, thus leading to burnout (Lawson et al., 2013). Therefore, firms and leaders must pay attention to WLB (Bataineh, 2019). Hypothesis 2 states the relationship between servant leadership and WLB. Certain studies indicate that servant leadership positively impacts WLB (Russo et al., 2016; Haar et al., 2017; Setyaningrum and Pawar, 2020; Utama et al., 2021). Previous studies have also proven that employees' WLB has a significant positive impact on innovative behavior (Arifin et al., 2021). The role of WLB as a mediator in organizational factors is important for practitioners seeking to improve their organization's performance (Stankviciene et al., 2021). Haar et al. (2017) finds that WLB mediates the impact of servant leadership on work engagement. However, no other studies have analyzed WLB as a mediator in the relationship between servant leadership and innovative behavior. According to the above-mentioned research, it can be predicted that WLB mediates the relationship between servant leadership and innovative behavior. Therefore, we propose the following relationship:

*Hypothesis 4*: Work–life balance has a mediating effect on the relationship between servant leadership and innovative behavior.

### Mediating Effect of Psychological Safety

Employees' psychological safety largely depends on the leadership behavior of the leader (Edmondson, 1999). Servant leadership holds that followers' trust in leaders and psychological safety can be enhanced by serving their needs, empowering them, empathizing with them, conceptualizing their skills, creating value for the community, prioritizing subordinates, behaving ethically, and helping them to grow and succeed (Liden et al., 2008; Carmeli et al., 2010; Krog and Govender, 2015; Kashyap and Rangnekar, 2016; Brohi et al., 2018). Employees are likely to adopt new practices and innovative behavior in a working environment with high psychological safety (Carmeli and Gittell, 2009; Gong et al., 2012; Cao and Zhang, 2019; Andersson et al., 2020). Iqbal et al. (2020) establishes that servant leadership has a direct and positive relationship with employees' innovative behavior. Moreover, psychological safety and thriving at work partially mediate this relationship. Chughtai (2016) reports that psychological safety partially mediates the effects of servant leadership on voice behavior. Wang et al. (2021) show that psychological safety potentially mediates the relationship between inclusive leadership and innovation. Carmeli et al.'s (2014) research reveals that psychological safety mediates the relationship between transformational leadership and creative problem-solving. In addition, members' psychological safety partially mediates the impact of leaders' servant leadership on innovative behavior. Therefore, we propose the following relationship:

*Hypothesis 5*: Psychological safety will have a mediating effect on the relationship between servant leadership and innovative behavior.

### Moderating Effect of Organizational Climate

Organizational climate is defined as “a set of measurable properties of the work environment perceived directly or indirectly by the people who live and work in this environment and assumed to influence their motivation and behavior” (Litwin and Stringer, 1968; Jafri et al., 2016). Organizational climate can also be defined as a set of underlying values, beliefs, and principles that employees perceive as held within their organization (Yoshida et al., 2013). Ötkena and Cenkcib (2015) divide organizational climate into three factors: humanistic climates, formalization climates, and risk-taking. This study also examines these three factors of organizational climate. Organizational climate dimensions, such as autonomy and freedom, positively influence innovative behavior (Shanker et al., 2017). Employees work in an environment where freedom is perceived to exist as they experience greater free will and take greater control of their ideas and work processes, thereby enhancing their innovativeness (Amabile et al., 1996; Si and Wei, 2012).

Servant leadership is a management style that provides services in harmony, whereby interaction with the environment exists (Trompenaars and Voerman, 2009). Khattak et al. (2017) finds that an organizational climate moderates the relationship between leadership style and employee creativity. An organization's creativity climate moderates the effects of leadership on employee creativity and workplace innovative orientation. Additionally, individual creativity can be enhanced in a risk-taking-supported culture (Ghosh, 2015). Organizational climate is crucial to enhance the quality of all the aspects of the innovation process, including the invention, development, and implementation of new ideas (Garud et al., 2013; Andersson et al., 2020). Therefore, we propose the following relationship:

*Hypothesis 6*: Organizational climate has a moderating effect on the relationship between servant leadership and innovative behavior.

**Figure 1:**
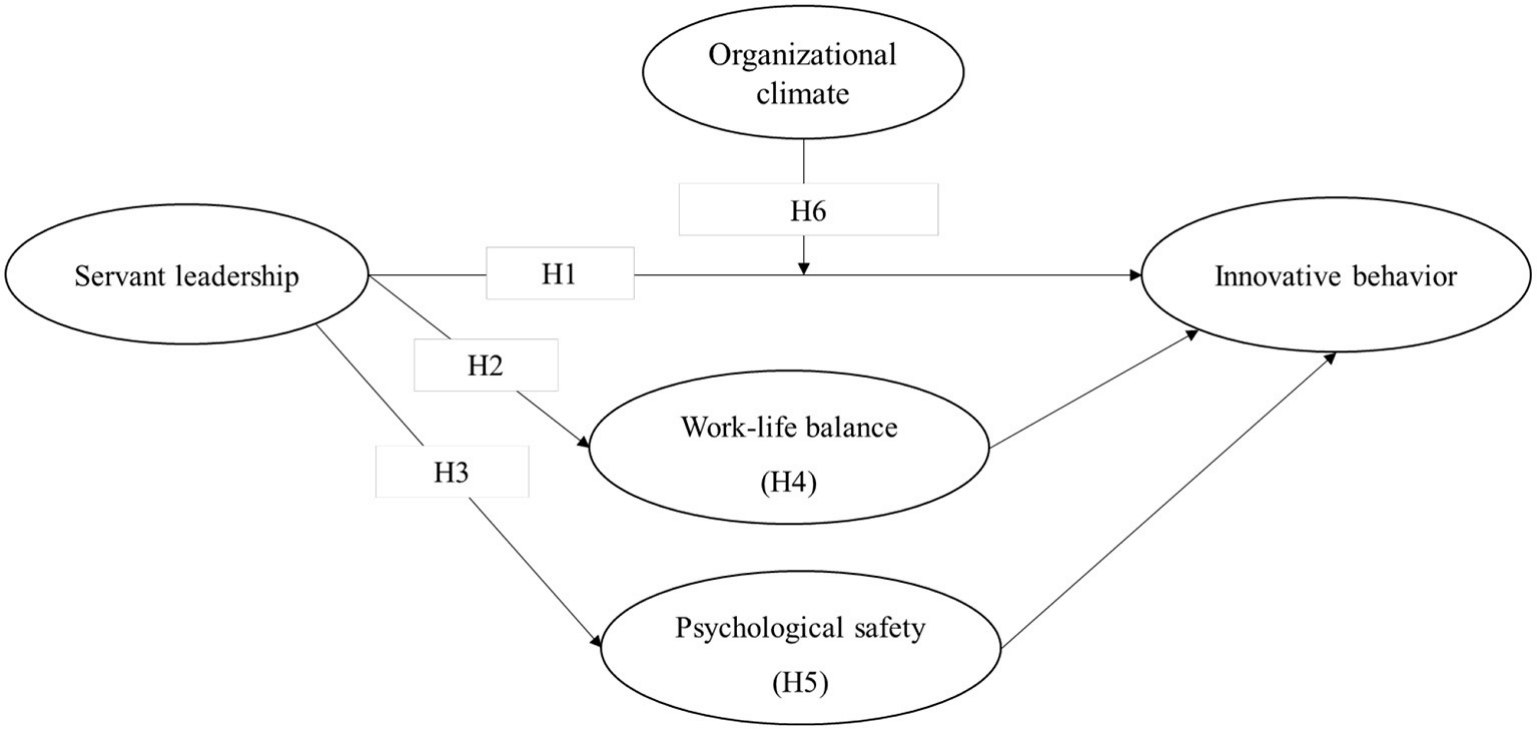
Research model.

The research model and proposed research hypotheses are depicted in Figure 1.

## Methods

### Sample and Data Collection

To empirically verify the effects of servant leadership on employees' innovative behavior, this study conducted a survey targeting employees of Chinese MNCs. More specifically, we empirically tested these ideas by examining how servant leadership shapes the innovative behavior of Chinese employees in these MNCs. In doing so, we collected data from a sample of 307 Chinese employees who were employed by five different Chinese MNCs from the Internet, IT, electronics, and e-commerce industries. Data were collected from August to September 2021 through an online survey. Because the sampled companies were very supportive and allowed employees to complete the survey during company time, 336 of 500 possible respondents participated. Twenty-nine questionnaires were excluded from the final analysis due to missing information, resulting in a final sample of 307 (67.2% response rate). The respondent sample profiles are summarized in Table 1. As shown in Table 1, 50.8% of the respondents were male, 42% were under 30 years old, and more than 64% had received a university degree. Furthermore, around 35% occupied sales and service positions, and 37% had less than seven years of experience in their present job. Overall, the sample can be described as employees who are relatively young, highly educated, and have a relatively short tenure at their present job.

**Table 1:** Sample profile.

**Sample characteristics**	***N***	***%***
*Gender*		
Female	151	49.2
Male	156	50.8
*Age (years)*		
Under 25	13	4.2
25–30	118	38.4
31–35	79	25.7
36–40	62	20.2
Over 40	35	11.4
*Education*		
High school	2	0.7
Applied university	22	7.2
Undergraduate	197	64.2
Graduate	86	28.0
*Tenure (years)*		
1–3	47	15.3
4–6	68	22.1
7–10	68	22.1
Over 10	53	17.3
*Position*		
Sales and service	106	34.5
Production	16	5.2
Administrative planning	37	12.1
R&D	71	23.1
Others	77	25.1
Total	307	100

As we use self-report questionnaires to collect data from the same participants, our data may suffer from potential common method variance (CMV) (Chang et al., 2010). To minimize the CMV concern inherent in our survey research, we take certain procedural and statistical steps. First, in the cover letter accompanying the questionnaires, we inform the respondents of their anonymity and confidentiality of their responses by emphasizing through that there are no “right” or “wrong” answers and the respondents are free to answer the questionnaires. Second, we carefully design the questionnaire by randomizing the order to the questions using survey software and reversed the scaling on several independent variable questions on the questionnaire. Notwithstanding these efforts reduce the potential for CMV, we check for CMV using Harmon's one-factor test after collecting the data. According to Podsakoff et al. (2003), high CMV is a serious problem in our data if a single factor emerges from the factor analysis, or one general factor accounts for most of the variance in the independent and criterion variables. We conduct the test by entering all self-reported variables into a factor analysis and examining the unrotated factor solution. The results yield six factors with eigenvalues greater than one, the first of which explains only 39% of the variation. Because no single dominant factor accounts for the majority of the covariance (i.e., more than 50%) among the self-reported variables, CMV is unlikely to be a serious problem in our data.

### Measures

To prepare for the questionnaire, we first develop the original English questionnaire and then translated survey items into Chinese with the assistance of two independent professional translators who are competent in both Chinese and English. To ensure the accuracy of the translation, we utilize a back-translation approach with two additional independent bilingual translators. Any conflicts are discussed by the researchers of this study and translators until they an agreement is reached (Hoskisson et al., 2000).

Unless otherwise indicated, we adapt all the scales used to measure the constructs of the study from established studies and measure all perceptual scales using 5-point Likert scales ranging from 1 (strongly disagree) to 5 (strongly agree). To measure employees' innovative behavior, we adapt six items from Scott and Bruce (1994). A sample item of the scale is: “I generate creative ideas at work.” To measure servant leadership, we adopt the five items developed by Liden et al. (2014). A sample item of the scale is: “My boss puts my best interests ahead of his/her own.” To measure WLB, we adapt five items from Haar (2013) and Valcour (2007). A sample item is: “I seem to enjoy every part of my life equally well.” We use Carmeli et al.'s (2010) five items to measure psychological safety at the individual level. A sample item includes: “No one in this organization would deliberately act in a way that undermines my efforts.” We capture organizational climate through nine items taken from Ötkena and Cenkcib (2015). A sample item of the scale includes: “Employees can easily access the information they need about the workflow.”

### Measurement Reliability and Validity Assessment

To assess the measurement reliability and validity, we conduct an exploratory factor analysis (EFA) followed by a confirmatory factor analysis (CFA). The EFA results are presented in Table 2. As shown in Table 2, the EFA results yield six factors, of which six items loaded on a single factor relating to innovative behavior; five items loaded on a second factor relating to WLB; six items loaded on third factor relating to the first dimension of organizational climate (HV: humanistic variance); five items loaded on a fourth factor describing servant leadership; three items loaded on a fifth factor relating to the second dimension of organizational climate (FV: formalization and risk-taking variance); and five items loaded on a sixth factor describing psychological safety. Therefore, these dimensions are kept separate in our subsequent analyses.

**Table 2:** Results of exploratory factor analysis.

**Items**	**Varimax rotation loadings (*****n*** **=** **307)**						**Percent variance explained**
	**Factor 1 (IB)**	**Factor 2 (WLB)**	**Factor 3 (OC: HV)**	**Factor 4 (SL)**	**Factor 5 (OC: FV)**	**Factor 6 (PS)**	
IB2	**0.858**	0.159	0.155	0.062	0.033	0.170	13.373
IB1	**0.770**	0.122	0.248	0.060	0.009	0.231	
IB6	**0.736**	0.179	0.112	0.165	0.198	0.040	
IB3	**0.704**	0.171	0.134	0.195	0.145	0.133	
IB5	**0.592**	0.117	0.208	0.195	0.398	0.060	
IB4	**0.539**	0.128	0.140	0.231	0.401	0.089	
WLB5	0.061	**0.866**	0.075	0.122	0.048	0.136	11.746
WLB3	0.173	**0.832**	0.113	0.099	0.239	0.065	
WLB4	0.161	**0.779**	0.072	0.195	0.039	0.188	
WLB2	0.128	**0.717**	0.131	0.192	0.138	0.081	
WLB1	0.310	**0.537**	0.201	0.112	0.108	0.115	
HV3	0.215	0.134	**0.683**	0.280	0.184	0.340	10.841
HV2	0.343	0.094	**0.650**	0.211	0.211	0.240	
HV1	0.312	0.120	**0.599**	0.283	0.167	0.079	
HV5	0.144	0.193	**0.599**	0.274	0.330	0.228	
HV4	0.150	0.196	**0.496**	0.373	0.291	0.103	
HV6	0.123	0.168	**0.433**	0.375	0.352	0.212	
SL4	0.197	0.153	0.242	**0.752**	0.127	0.170	10.358
SL2	0.074	0.113	0.121	**0.672**	0.144	0.087	
SL3	0.147	0.204	0.167	**0.596**	0.067	0.128	
SL1	0.128	0.149	0.245	**0.553**	0.134	0.253	
SL5	0.189	0.124	0.369	**0.463**	0.005	0.179	
FV3	0.145	0.169	0.172	0.077	**0.696**	0.180	8.380
FV2	0.270	0.122	0.322	0.158	**0.636**	0.225	
FV1	0.206	0.178	0.399	0.237	**0.530**	0.292	
PS4	0.169	0.226	0.361	0.266	0.208	**0.646**	7.463
PS3	0.201	0.245	0.372	0.247	0.136	**0.563**	
PS2	0.197	0.134	0.165	0.281	0.240	**0.526**	
PS5	0.165	0.166	0.129	0.160	0.425	**0.490**	
PS1	0.353	0.226	0.227	0.244	0.298	**0.440**	

*Loadings on a relevant factor are shown in bold and shaded in dark gray. SL, servant leadership; WLB, work–life balance; PS, psychological safety; IB, innovative behavior; OC, organizational climate; HV, humanistic variance; FV, formalization and risk-taking variance*.

We also assess the construct reliability and validity of our perceptual measures by estimating an overall six-factor CFA. The CFA results are shown in Table 3. As expected, the model provides a satisfactory fit to the data [$\chi_{\left(390\right)}^{2}$ = 922.82, *p* < 0.01 comparative fit index (CFI) = 0.94, Tucker–Lewis index (TLI) = 0.90, incremental fit index (IFI) = 0.94, root mean square error of approximation (RMSEA) = 0.05] (Anderson and Gerbing, 1988). Furthermore, all the factor loadings are highly significant (*p* < 0.001), and both the coefficient alpha values (0.832–0.901) and the composite reliabilities (0.837–0.904) of all the constructs exceed the 0.70 benchmark. All the average variances extracted (AVE) are >0.50. Therefore, our measures demonstrate adequate convergent validity and reliability (Fornell and Larker, 1981). To assess discriminant validity, we follow Fornell and Larker's (1981) procedure to compare the shared variance between all the possible pairs of constructs to determine whether they are lower than the AVE of the individual constructs. As shown in Table 4, the square root of the AVE of each construct is much higher than its highest shared variance with the other constructs, providing strong support for discriminant validity for all the constructs in the study.

**Table 3:** Results of reliability and validity assessment using confirmatory factor analysis.

**Construct and indicators**	**FL**
*Servant leadership (alpha = 0.832, CR = 0.837, AVE = 0.509)*
My leader would not compromise ethical principles to achieve success.	0.706
My leader gives me the freedom to handle difficult situations in the way that I feel is best.	0.669
My leader puts my best interests ahead of his/her own.	0.670
I would seek help from my leader if I had a personal problem.	0.848
My leader makes my career development a priority.	0.658
*Work–life balance (alpha = 0.899, CR = 0.902, AVE = 0.650)*
I seem to enjoy every part of my life equally well.	0.647
I am satisfied with my work–life balance, enjoying both roles.	0.783
I manage to balance the demands of my work and personal/family life well.	0.883
I manage to divide attention on work and personal/family life well.	0.832
I manage to divide time to work and personal/family life well.	0.865
*Psychological safety (alpha = 0.863, CR = 0.864, AVE = 0.562)*
I can bring up problems and tough issues.	0.753
It is safe to take a risk in this organization.	0.693
It is easy for me to ask other members of this organization for help.	0.788
No one in this organization would deliberately act in a way that undermines my efforts.	0.836
People in this organization sometimes reject others for being different (r)	0.664
*Organizational climate (alpha = 0.891, CR = 0.899, AVE = 0.597)*
Employees can easily access the information they need about the workflow.	0.727
This organization is usually open to new ideas, technologies, and applications.	0.804
Employees have good relationships based on mutual trust.	0.839
Senior management expects that all employees participate in decision-making processes related to their	0.731
work.	
Employees have some degree of freedom in planning and executing their work.	0.807
Bureaucratic formalities are in its minimum possible level.	0.720
*Organizational climate (alpha = 0.844, CR = 0.842, AVE = 0.642)*
There is high formalization and strict rules in the execution of work activities (r)	0.849
In general, this organization avoids taking risk when conducting business activities (r)	0.841
In general, work processes are monotonous and routine (r)	0.706
*Innovative behavior (alpha = 0.901, CR = 0.904, AVE = 0.613)*
I search out new technologies, processes, techniques, or product ideas	0.805
I generate creative ideas	0.858
I promote and champion ideas to others	0.792
I investigate and secure funds needed to implement new Ideas.	0.694
I develop adequate plans and schedule for the implementation of new ideas.	0.739
I consider myself innovative.	0.800

*N = 307. AVE, average variance extracted; CR, composite reliability; FL, factor loading. Model Summary: χ2 (390) = 922.82, p <0.01, CFI = 0.91, TLI = 0.90, IFI = 0.91, RMSEA = 0.06*.

**Table 4:** Descriptive statistics, correlations, and discriminant validity.

**Variable**	**Mean**	**SD**	**1**	**2**	**3**	**4**	**5**	**6**	**7**	**8**	**9**
1. Gender	1.508	0.501	–								
2. Age	2.961	1.102	−0.130^*^	–							
3. Education	3.195	0.584	−0.073	−0.024	–						
4. SL	3.512	0.763	−0.081	−0.009	−0.020	*0.714*					
5. WLB	3.741	0.736	0.013	0.062	0.106	0.428^**^	*0.806*				
6. PS	3.770	0.626	−0.085	0.045	0.073	0.599^**^	0.504^**^	*0.749*			
7. OC: HV	3.711	0.697	−0.133^*^	0.043	0.074	0.665^**^	0.453^**^	0.716^**^	*0.773*		
8. OC: FV	3.686	0.696	−0.091	0.102	0.017	0.479^**^	0.414^**^	0.663^**^	0.679^**^	*0.801*	
9. IB	3.754	0.651	−0.163^**^	0.071	0.106	0.449^**^	0.433^**^	0.573^**^	0.584^**^	0.539^**^	*0.783*

*N = 307. SL, servant leadership; WLB, work–life balance; PS, psychological safety; OC, organizational climate; HV, humanistic variance; FV, formalization and risk-taking variance; IB, innovative behavior. Figures in italics denote the square root of the AVE of each study construct*.

* *p <0.05*,

** *p <0.01*.

## Analyses and Results

### Hypothesis Testing Using Baron-Kenny “Three-Steps and Sobel” Approach

We present the basic descriptive statistics and correlations of the measures in Table 4. As expected, all the independent variables are correlated with their corresponding dependent variables. Servant leadership is significantly correlated with WLB, psychological safety, and innovative behavior.

To examine the role of servant leadership in predicting the WLB, psychological safety, and innovative behaviors of employees in Chinese MNCs, we employ a regression approach and report the results in Table 5. As shown in Models 2, 1, and 4 of Table 5, we find that servant leadership is positively and significantly related to innovative behavior (β = 0.442, t = 8.743, *p* < 0.001), WLB (β = 0.436, t = 8.467, *p* < 0.001), and psychological safety (β = 0.599, t = 13.055, *p* < 0.001), respectively, thus providing strong support for Hypotheses 1, 2, and 3, respectively. To test the extent to which WLB and psychological safety mediate the influence of servant leadership on innovative behaviors, we employ the three-up mediated regression approach recommended by Baron and Kenny (1986) and report the results in Table 5. To test the first mediation condition, we test the effect of servant leadership on WLB and psychological safety, and the results shown in Models 1 and 4 of Table 5 demonstrate that servant leadership is positively and significantly related to both WLB (β = 0.436, t = 8.467, *p* < 0.001) and psychological safety (β = 0.599, t = 13.055, *p* < 0.001), thus satisfying the first mediation condition (Baron and Kenny, 1986, p. 1176). To test the second mediation condition, we estimate a new model that specifies only the direct relationship between servant leadership and the two mediators (WLB and psychological safety). Models 2 and 5 of Table 5 show that without the presence of the WLB and psychological safety mediators, servant leadership is positively and significantly related to innovative behavior (β = 0.442, t = 8.743, *p* < 0.001). These results satisfy the second mediation condition. Finally, after entering the mediators of WLB and psychological safety, the results shown in Models 3 and 4 of Table 5 indicate that WLB and psychological safety are both significantly related to innovative behavior. Focusing on these results, we establish that servant leadership still positively and significantly affects innovative behaviors in the presence of the mediators of both WLB and psychological safety. These findings demonstrate that WLB and psychological safety partially mediate the effect of servant leadership on innovative behavior. We also find that there is a substantial reduction, though still significant, in the coefficient for the direct effect of servant leadership on innovative behaviors after entering the mediators of WLB (from 0.442 to 0.316) and psychological safety (from 0.442 to 0.169). Thus, WLB and psychological safety both partially mediate the relationship between servant leadership and innovative behavior. We further perform the Sobel test to empirically examine the significance of the possible indirect effects of servant leadership on innovative behaviors through WLB and psychological safety. Since the Sobel test can examine the combined effects of both the mediating variable's effect on the dependent variable and the effect of the independent variable on the mediating variable, this test is a more direct approach to empirically test for the mediation hypotheses. As shown in Table 5, we find that the independent variable, servant leadership, has a positive and statistically significant indirect effect *via* WLB (z_value_ = 4.506, *p* < 0.001) and psychological safety (z_value_= 6.750, *p* < 0.001) on innovative behaviors, thereby leading to strong support for Hypotheses 4 and 5.

**Table 5:** Results for regression analyses with potential mediating effects.

**Variables**	**Model 1 (WLB)**	**Model 2 (IB)**	**Model 3 (IB)**	**Sobel test**	**Model 4 (PS)**	**Model 5 (IB)**	**Model 6 (IB)**	**Sobel test**
Gender	0.067 (1.288)	−0.111^*^ (−2.165)	−0.130^**^ (−2.651)		−0.024 (−0.511)	−0.111^*^ (−2.165)	−0.100^*^ (−2.142)	
Age	0.078 (1.502)	0.063 (1.236)	0.040 (0.826)		0.049 (1.061)	0.063 (1.236)	0.041 (0.873)	
Education	0.122* (2.360)	0.108* (2.135)	0.073 (1.491)		0.085 (1.848)	0.108 (2.135)	0.069 (1.494)	
Servant leadership	0.436^***^ (8.467)	0.442^***^ (8.743)	0.316^***^ (5.869)	4.506^***^	0.599^***^ (13.055)	0.442^***^ (8.743)	0.169^***^ (2.928)	6.750^***^
Work-life balance			0.289^***^ (5.349)					
Psychological safety							0.457^***^ (7.888)	
*R* ^2^	0.205	0.232	0.299		0.369	0.232	0.364	
*F* statistics	19.474^***^	22.867^***^	25.688^***^		44.173^***^	22.867^***^	34.447^***^	

*N = 307. WLB, work–life balance; PS, psychological safety; IB, innovative behavior. The figures shown in the table are standardized values with t-statistics in parentheses*.

* *p <0.05*,

** *p <0.01*,

*** * p <0.001*.

### Hypothesis Testing Using a Moderated Hierarchical Regression Analyses

Because our hypotheses suggest interaction terms composed of servant leadership and two subdimensions of organizational climate, we employ a moderated regression analysis to empirically test our hypotheses, which are deemed appropriate for testing the effects (Aiken and West, 1991). Thus, we adopt a moderated hierarchical approach, wherein we first include the control variables in the mode, then add the focal variables, and finally include the interaction terms. To minimize possible multicollinearity between the interaction terms and their components, we follow Aiken and West's (1991) recommendation by mean-centering each scale that constitutes an interaction term and creating the interaction terms by multiplying the relevant mean-centered scales. We also check for the potential multicollinearity problem by examining the variance inflation factor (VIF). Because the largest VIF in the models is 2.47, which is well below the accepted threshold of the benchmark of 10.0, multicollinearity is not a serious concern in our analysis.

The hierarchical procedure of the moderated regression analysis results in four models, labeled Models 1–4, which are reported in Table 6. In Model 1 of Table 6, we address the role of servant leadership and, consistent with our prediction in Hypothesis 1, servant leadership is positively and significantly related to innovative behaviors. With Hypothesis 6, we consider the moderating role of organizational climate. In Models 2 and 3 of Table 6, we assess the moderating role of organizational climate by examining the interactive effects between servant leadership and two specific subdimensions of organizational climate, that is, humanistic variance (HV), formalization and risk-taking variance (FV), on innovative behaviors. As shown in Model 2 of Table 6, the interaction between servant leadership and HV is positively associated with innovative behavior (β = 0.164, t = 3.689, *p* < 0.001). Similarly, we find that the interaction between servant leadership, and FV is positively associated with innovative behavior. Therefore, Hypothesis 6 is strongly supported. The results of the full model (Model 6) are presented in Table 6, which includes all the independent variables of interest and interaction terms, thus suggesting that the results are robust. With all the variables entered into the model, all but one coefficient that are statistically significant in the earlier models remain statistically significant in the full model. As shown in Model 4 of Table 6, the moderating impact of HV on the relationship between servant leadership and innovative behaviors loses its significance in the full model. This mixed result for the moderated impact of HV can be explained by the fact that the correlation between the two subdimensions of organizational climate (i.e., HV and FV) is highly significant, indicating a potential problem that leads to an understanding of the impact of HV on innovative behavior.

**Table 6:** Results of the moderated regression analysis.

**Variables**	**Model 1**	**Model 2**	**Model 3**	**Model 4**
Gender	−0.113^*^ (2.451)	−0.093^*^ (−2.107)	−0.106^*^ (−2.428)	−0.094^*^ (−2.183)
Age	0.030 (0.660)	0.026 (0.586)	0.011 (0.255)	0.012 (0.291)
Education	0.054 (1.170)	0.063 (1.428)	0.086 (1.966)	0.075 (1.744)
Work–life balance	0.173^**^ (3.216)	0.124^*^ (2.370)	0.091 (1.744)	0.084 (1.621)
Psychological safety	0.392^***^ (6.480)	0.272^***^ (4.127)	0.297^***^ (4.521)	0.232^**^ (3.388)
Servant leadership (SL)	0.132^*^ (2.282)	0.010 (0.167)	0.080 (1.453)	0.008 (0.143)
Humanistic variance (HV)		0.324^***^ (4.673)		0.222^**^ (3.056)
Formalization variance and risk taking (FV)			0.299^***^ (5.042)	0.229^**^ (3.620)
SL × HV		0.164^***^ (3.689)		0.004 (0.047)
SL × FV			0.220^***^ (4.850)	0.216^**^ (2.755)
*R* ^2^	0.385	0.445	0.459	0.476
Δ *R*^2^		0.060^***^	0.074^***^	0.091^***^
*F* statistics	31.320^***^	29.927^***^	31.656^***^	26.901^***^

*The figures shown in the table are standardized values with t-statistics in parentheses*.

* *p <0.05*,

** *p <0.01*,

*** *p <0.001*.

### Supplementary Analysis

To ensure the robustness of the results reported in this study, we perform robustness tests by applying partial least squares structural equation modeling (SEM). Consistent with the two-step modeling approach, we estimate a measurement model prior to examining the structural model relationships (Chin, 1998). We perform various tests to assess the reliability and validity of the measurement model and present the results in Table 7. As shown in Table 7, the composite reliabilities are all >0.80 (from 0.883 to 0.926), thus exceeding the threshold of 0.70. These results provide evidence of internal consistency (Fornell and Larker, 1981).

**Table 7:** Measurement reliability and validity assessments using structural equation modeling.

**Construct and indicators**	**FL**
*Servant leadership (CR = 0.883, AVE = 0.602, square roots of AVE = 0.776)*	
My leader would not compromise ethical principles to achieve success.	0.777
My leader gives me the freedom to handle difficult situations in the way that I feel is best.	0.743
My leader puts my best interests ahead of his/her own.	0.749
I would seek help from my leader if I had a personal problem.	0.869
My leader makes my career development a priority.	0.732
*Work–life balance (CR = 0.925, AVE = 0.713, square roots of AVE = 0.844)*	
I seem to enjoy every part of my life equally well.	0.764
I am satisfied with my work–life balance, enjoying both roles.	0.840
I manage to balance the demands of my work and personal/family life well.	0.895
I manage to divide attention on work and personal/family life well.	0.852
I manage to divide time to work and personal/family life well.	0.863
*Psychological safety (CR = 0.901, AVE = 0.647, square roots of AVE = 0.804)*	
I can bring up problems and tough issues.	0.815
It is safe to take a risk in this organization.	0.780
It is easy for me to ask other members of this organization for help.	0.828
No one in this organization would deliberately act in a way that undermines my efforts.	0.857
People in this organization sometimes reject others for being different (r)	0.736
*Organizational climate (CR = 0.922, AVE = 0.662, square roots of AVE = 0.814)*	
Employees can easily access the information they need about the workflow.	0.799
This organization is usually open to new ideas, technologies, and applications.	0.853
Employees have good relationships based on mutual trust.	0.854
Senior management expects that all employees participate in decision-making processes related to their	0.788
work.	
Employees have some degree of freedom in planning and executing their work.	0.833
Bureaucratic formalities are in its minimum possible level.	0.752
*Organizational climate (CR = 0.905, AVE = 0.761, square roots of AVE = 0.872)*	
There is high formalization and strict rules in the execution of work activities (r)	0.879
In general, this organization avoids taking risk when conducting business activities (r)	0.910
In general, work processes are monotonous and routine (r)	0.827
*Innovative behavior (CR = 0.926, AVE = 0.675, square roots of AVE = 0.822)*	
I search out new technologies, processes, techniques, or product ideas	0.812
I generate creative ideas	0.861
I promote and champion ideas to others	0.823
I investigate and secure funds needed to implement new Ideas.	0.780
I develop adequate plans and schedule for the implementation of new ideas.	0.817
I consider myself innovative.	0.834

*N = 307. AVE, average variance extracted; CR, composite reliability; FL, factor loading*.

Moreover, the outer loadings of all constructs are loaded strongly and significantly on their respective factors, and the values are greater, ranging from 0.732 to 0.910, thus indicating convergent validity and reliability. The AVE values for all the constructs are all above the 0.5 cutoff (from 0.602 to 0.761), demonstrating adequate convergent validity for the measures (Fornell and Larker, 1981). In addition, we compare the square root of the AVE of each construct, which is higher than the correlations between the constructs and others, thus suggesting adequate discriminant validity for the measures (Fornell and Larker, 1981). The comparison of the loading values of each indicator with the cross-loadings with other indicators indicates that each indicator loading is higher than the respective cross-loadings, again demonstrating adequate discriminant validity.

In addition, following prior work (Stone, 1974; Geisser, 1975), we assess the predictive validity of the latent constructs in the model using Stone–Geisser's Q2, and we verify that both the cross-validated communality and redundancy values are all higher than zero, suggesting the presence of predictive validity in the model (Fornell and Cha, 1994).

**Figure 2:**
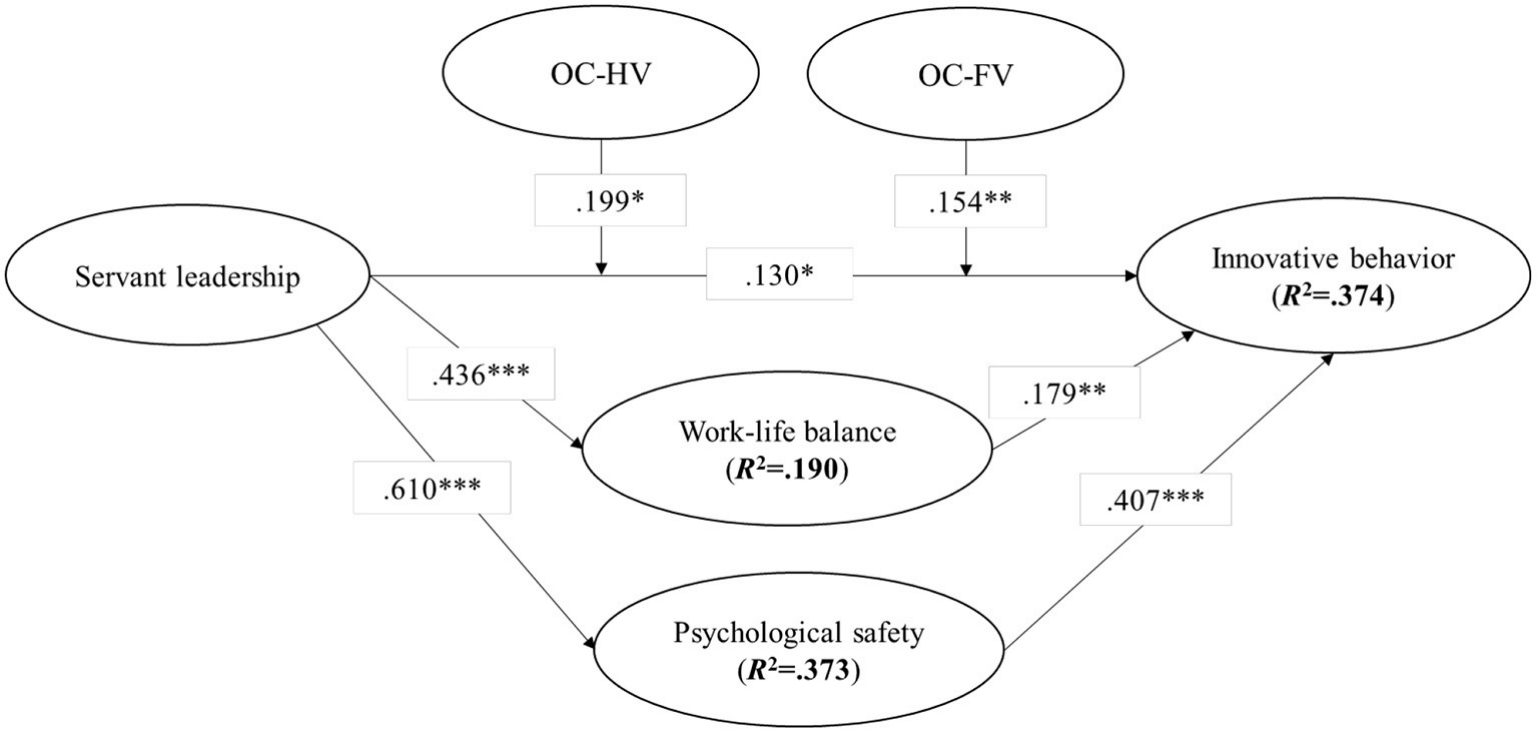
Results of structural equation modeling. *N* = 307. OC, organizational climate; HV, humanistic variance; FV, formalization variance and risk-taking variance; n.s., non-significant. *p < 0.05, **p < 0.01, ***p < 0.001.

Figure 2 shows the results of the structural equation model. As shown in Figure 2, the results indicate a significant positive relationship between servant leadership and innovative behaviors (*b* = 0.130, *p* < 0.05), WLB (*b* = 0.436, *p* < 0.001), and psychological safety (*b* = 0.610, *p* < 0.001), thus supporting Hypotheses 1, 2, and 3, respectively. To assess the potential of WLB and psychological safety in mediating the effect of servant leadership on innovative behaviors, we examine the direct link between servant leadership and innovative behavior. When we exclude the possible mediator variables (i.e., WLB and psychological safety), the original result for the effect of servant leadership still holds for the mediator-without model. In addition, the *R*^2^ of innovative behavior in the mediator-without model is lower than that in the original full model. Altogether, these results suggest that WLB and psychological safety play an important role in partially mediating the effect of servant leadership on innovative behavior. Finally, we follow Zhao et al.'s. (2010) procedure for estimation mediation, test the potential indirect effects in our model, and present the results in Table 8. As reported in Table 8, all the indirect effects in the research model are statistically significant at least at the 0.01 level, again demonstrating that WLB and psychological safety both play a role in partially mediating the effect of servant leadership on innovative behavior. Finally, we test Hypothesis 6 by examining the possible role of organizational climate in moderating the relationship between servant leadership and innovative behavior. As shown in Figure 2, the path coefficient of the interaction term between servant leadership and the subdimension of organizational climate, namely, HV, is positive and statistically significant (*b* = 0.199, *p* < 0.05). Similarly, the path coefficient of the interaction of servant leadership and the other subdimension of organizational climate, that is, FV is also positive and significant (*b* = 0.194, *p* < 0.01). Taking these results into consideration, the results provide strong support for Hypothesis 6. Overall, the results of the SEM are robust to the use of the regression method.

**Table 8:** Results of structural model assessment for direct and indirect effects.

**Effect**	**Estimate**	***T*-values**	***P*-values**
*Direct effects*			
Servant leadership → Work–life balance	0.436	10.364	^***^
Servant leadership → Psychological safety	0.610	16.286	^***^
Servant leadership → Innovative behavior	0.130	2.033	^*^
Work–life balance → Innovative behavior	0.179	3.247	^**^
Psychological safety → Innovative behavior	0.407	6.689	^***^
*Indirect effects*			
Servant leadership → Work–life balance → Innovative behavior	0.078	3.095	^**^
Servant leadership → Psychological safety → Innovative behavior	0.249	6.794	^***^

* *p <0.05*,

** *p <0.01*,

*** *p <0.001*.

## Discussion and Conclusions

This study is conducted to determine the impact of servant leadership on innovative employee behavior in Chinese MNCs. It also aims to establish whether the WLB and psychological safety of employees and the degree of organizational climate mediate and moderate, respectively, the relationship between servant leadership and employees' innovative behavior.

First, we find that servant leadership plays a vital role in enhancing employees' innovative behaviors. This has also been verified by other studies (Karatepe et al., 2020). Understanding how leadership styles promote innovative behavior has become an important research question in innovation management (Melroy et al., 2015; Nadolna, 2020). Leadership plays a decisive role in enhancing organizational creativity as well as launching and driving innovation projects (Stoker et al., 2001; Mumford et al., 2002; Bossink, 2007; Kesting et al., 2015), especially servant leadership.

Second, we also find that servant leadership positively and significantly affects WLB and psychological safety. This means that servant leaders can provide their followers with greater opportunities to balance their work and life roles (Haar et al., 2017). Moreover, servant leaders also emphasize that the concepts of “service” and “altruism,” center on employees, trust subordinates, and establishing good relationships with them. When employees encounter difficulties, servant leaders can address the behavior of their subordinates tolerantly, fairly, and impartially. They can reduce their perceptions of the risks of pro-social violations and improve their psychological safety (Brohi et al., 2018).

Third, WLB and psychological stability mediate the relationship between servant leadership and employees' innovative behaviors. Few studies have explored the psychological safety between servant leadership and employees' innovative behaviors and the mediating role of WLB. Previous studies have discussed the intermediary effect of psychological safety between inclusive leadership and employee involvement in creative tasks in the workplace (Carmeli et al., 2010), transformational leadership and creative problem-solving (Carmeli et al., 2014), participative leadership, and employee creativity (Chen et al., 2020). However, this study emphasizes the intermediary effect of psychological safety between service-oriented leadership and employee innovative behavior. This will provide theoretical support for many scholars studying servant leadership, psychological safety, and WLB.

Finally, organizational climate moderates the relationship between servant leadership and employee innovative behavior. Organizational climate is very important for improving innovation, including invention development and implementation of new ideas (Andersson et al., 2020). Leaders should apply servant leadership styles to improve employee and organizational creativity (Wang and Rode, 2010). They should create an environment that supports innovation, by encouraging both humanistic and innovative climates.

### Managerial Implications

Based on the results of this study, leaders are recommended to apply servant leadership styles to improve employees' innovative behaviors. With the onset of the COVID-19 pandemic, employees have become psychologically anxious, experienced deteriorating working conditions, and developed a fear of losing jobs. In this study, we conduct theoretical and empirical discussions on WLB and psychological safety, which are the main concerns of employees today. Managers can provide WLB support to employees through servant leadership, thereby allowing employees to balance work and life effectively. In addition, managers' service and support can improve employees' psychological security, thereby enhancing their innovative behavior and realizing the enterprise's innovative performance.

Regarding organizational climate, managers should provide employees with diverse and flexible working environments and provide adequate adventurous support. Thus, employees can easily access the information they need about the workflow and be open to new ideas, technologies, and applications. Employee performance is the main criterion for evaluating the reward mechanism. Employees ought to have some degree of freedom in planning and executing their work. Thus, this study contributes to the research on international human resource management by offering important implications for MNCs on how to effectively improve the management of their global workforce to respond to the dramatically changing global landscape in the post-COVID-19 era.

### Limitation

This study has certain limitations. First, we use only a single sample from Chinese MNCs. Different samples would enhance the understanding of cross-level processes among employees, such as innovative behaviors and servant leadership in MNCs. China's diverse perspectives and cultural values, such as collectivism and high power distance, differ from those of other countries (Chen et al., 2020). Thus, future research should use more samples of MNCs from other economies for comparative analysis and reflect on the effects of leadership on employee innovation behavior under different cultural backgrounds. Moreover, with scholars from different countries, such research should expand the scope of the investigation and add cross-cultural, diversity management, and other related variables to improve this research. In addition, although the article proposes that changes in the work environment and work style during the epidemic will also have a certain impact on psychological safety and work–family balance, this article does not specifically set variables related to the work environment or work style, for example, working offline or online, working at home, or working in the workplace. Therefore, the burning question is: “How does the support provided by servant leadership have different effects on employees' psychological safety and employee innovation?”

## Data Availability Statement

The raw data supporting the conclusions of this article will be made available by the authors, without undue reservation.

## Ethics Statement

The studies involving human participants were reviewed and approved by Gachon University Ethics Committee. The patients/participants provided their written informed consent to participate in this study.

## Author Contributions

All authors listed have made a substantial, direct, and intellectual contribution to the work and approved it for publication.

## Funding

This work was supported by the Gachon University Research Fund of 2021 (GCU-202103510001).

## Conflict of Interest

The authors declare that the research was conducted in the absence of any commercial or financial relationships that could be construed as a potential conflict of interest.

## Publisher's Note

All claims expressed in this article are solely those of the authors and do not necessarily represent those of their affiliated organizations, or those of the publisher, the editors and the reviewers. Any product that may be evaluated in this article, or claim that may be made by its manufacturer, is not guaranteed or endorsed by the publisher.
